# Efficacy of Probiotics Compared to Chlorhexidine Mouthwash in Improving Periodontal Status: A Systematic Review and Meta-Analysis

**DOI:** 10.1155/2023/4013004

**Published:** 2023-01-23

**Authors:** Kaio Henrique Soares, Parsa Firoozi, Glaciele Maria de Souza, Olga Beatriz Lopes Martins, Saulo Gabriel Moreira Falci, Cassio Roberto Rocha dos Santos

**Affiliations:** ^1^Department of Dentistry, School of Biological and Health Sciences, Universidade Federal dos Vales do Jequitinhonha e Mucuri, Rua da Glória,N. 187 Centro, Diamantina, Minas Gerais, Brazil; ^2^Student Research Committee, School of Dentistry, Zanjan University of Medical Sciences, Zanjan, Iran

## Abstract

**Purpose:**

To critically evaluate the available literature and conduct a systematic review of recent randomized controlled trials to assess the effectiveness of probiotics compared to chlorhexidine mouthwash in enhancing periodontal health.

**Methods:**

Five databases were searched electronically, as well as the gray literature. Using the Cochrane risk-of-bias tool for randomized clinical trials, the risk of bias was examined. The weighted mean difference (WMD) method was used to calculate the effect sizes. Heterogeneity was assessed using *I*^2^ and *τ*^2^ statistics. The GRADE approach was adopted to assess the certainty of the evidence. To assess the robustness of the findings, sensitivity analysis and publication bias assessment were undertaken.

**Results:**

A total of 1850 studies were initially identified. Sixteen clinical trials were eligible for qualitative synthesis, and ten were included in the meta-analysis. In terms of the gingival index, in total, no statistically significant difference was observed between chlorhexidine and probiotics within 4 weeks (WMD −0.03, 95% CI: −0.09∼0.04, *P* = 0.3885). Similar to GI, no statistically significant difference was observed between chlorhexidine and probiotics regarding the plaque index within 4 weeks (WMD 0.11, 95% CI: −0.05∼0.28, *P* = 0.1726). No statistically significant difference was observed between chlorhexidine and probiotics in all time intervals regarding oral hygiene index-simplified (WMD −0.01, 95% CI: −0.05∼0.04, *P* = 0.7508). The robustness of these findings was confirmed by sensitivity analysis and publication bias assessments.

**Conclusions:**

Based on the findings, probiotics were an acceptable alternative to conventional chlorhexidine in improving periodontal health. High-quality studies with rigorous methodology should be conducted to assess the optimum doses of probiotics for clinical implications.

## 1. Introduction

One of the most prevalent oral diseases in the world is periodontal disease [1]. Dental plaque has been proved to be the main factor in the onset and progression of periodontal diseases. Thus, the prevention and treatment of periodontal diseases are based on plaque control and antimicrobial therapies [2].

Adjuvant chemical approaches, such as mouthwash, have been proposed as an additional therapy due to the limitations of mechanical plaque management techniques and the rise in antibiotic resistance. [3].

Antimicrobial agents such as hydrogen peroxide, chlorhexidine (CHX), essential oils, cetylpyridinium chloride (CPC), and triclosan are commonly used for this purpose. Based on the present findings, the gold standard for plaque control is chlorhexidine, a broad-spectrum antimicrobial agent [2]. However, continuous use of chlorhexidine has side effects such as an increase in calculus formation, alteration in taste, and oral mucosal erosion [4].

Probiotic usage has been suggested as an alternative to manage periodontal diseases in recent years. In this way, probiotics may be a turning point in periodontal treatment [5]. They were defined as “living microorganisms that, given in sufficient quantities, bring health benefits to the host” [5]. Probiotics have been shown to reduce the acidic pH inside the oral cavity and release bacteriocins that prevent plaque formation [6].

Moreover, previous studies have shown that the use of probiotic products can reduce oral caries. In these studies, reducing the level of some bacteria effective in causing caries, including *Streptococcus mutans*, has been shown [7]. Probiotics have also been reported to be associated with decreased *Candida* colonies in saliva and the prevalence of oral candidiasis. By inhibiting the growth of microorganisms, probiotics can change the host's microbiome [8].

The role of probiotics in periodontal disease and a significant decrease in plaque indices, bleeding on probing, and gingivitis have been proposed [9]. However, only a small number of clinical trials have examined the antiplaque and anti-inflammatory effects of probiotics and chlorhexidine mouthwash. This systematic review and meta-analysis aimed to compare the use of chlorhexidine mouthwash and oral probiotics to evaluate the efficacy of probiotics as a potential alternative agent for the improvement of periodontal status and critically appraise the available literature.

## 2. Materials and Methods

### 2.1. Protocol and Registration

This review was organized based on the Preferred Reporting Items for Systematic Reviews and Meta-Analysis (PRISMA) guidelines [10]. Also, it was registered at PROSPERO with the protocol registration code: CRD42021261054.

### 2.2. Eligibility Criteria

The main objective of this meta-analysis was to respond to the following question: Do probiotics have the same periodontal health-improving power as CHX mouthwash?

The PICO components were the following items: population (any patients with no restrictions regarding their age), intervention (probiotics in any form or type), comparator (conventional chlorhexidine mouthwash), outcome (clinical parameters such as the plaque index, the gingival index, the probing pocket depth, the clinical attachment level, the sulcular bleeding index, gingival recession, and the periodontal inflamed surface area index), and study design (randomized controlled trials). No language restriction was applied to decrease the risk of language bias. Studies were excluded if they were nonpeer-reviewed RCTs and conference papers, editorial, and review papers.

### 2.3. Information Sources and Search

The electronic search for articles took place in December 2021. The following databases were searched: PubMed, Web of Science, Scopus, EMBASE, Virtual Health Library, Cochrane Oral Health, and Group Trial Register. The gray literature was also searched through Google Scholar. The MeSH (Medical Subject Headings) database was utilized to verify all keywords (Supplementary Table 1). Free relevant keywords were searched additionally. Moreover, all primary research' reference lists were thoroughly searched for further scientific papers.

### 2.4. Study Selection

Two reviewers (KHS and OBLM) rigorously and impartially evaluated the articles. Reading through all of the article titles and abstracts in the aforementioned databases served as the first step in the selection process. Articles that did not meet the predefined inclusion criteria were excluded after reading the titles and abstracts. The full texts of the papers that were retrieved after the first step were gathered, and full-text publications were evaluated by the authors following inclusion criteria. Any discrepancies concerning eligibility and any disputes between the two reviewers were settled by discussion.

### 2.5. Data Extraction

The data were extracted by one author, and a second reviewer subsequently double-checked the data. All the data obtained by the articles were tabulated as follows: study design, sample size, age of participants, experimental groups, comparator groups, clinical parameters, and follow-ups. In addition, if studies had insufficient data for the meta-analysis, the authors were contacted to provide them.

### 2.6. Risk of Bias

Two reviewers (GMS and KHS) independently evaluated the risk of bias in this review using the Cochrane Collaboration's assessment tool (version 2) [11]. The randomization procedure, variations from intended interventions, missing outcome data, assessment of the outcome, and selection of the reported result are the five primary domains of this tool.

### 2.7. Data Analysis

The meta-analysis was conducted on the gingival index, plaque index, and oral hygiene index-simplified (OHI-S). The “meta” package and the R software, version 3.6.2, were used for all of the analysis. If the data were numerically similar, the weighted mean difference (WMD) utilizing the inverse variance approach was examined for continuous variables. Apart from that, the standard mean difference (SMD) was used. The fixed-effect model was used when *I*^2^ = 0, and the random-effect model was used when *I*^2^ > 0. All the *P* values were two-sided, and the statistical significance was defined at a level of *α* = 0.05. The same methodology used in three previously published meta-analyses was adopted [12–14]. The leave-one-out method, which recalculates the meta-analysis*N* − 1 time while omitting one study each time, was used for sensitivity analysis. Outliers can be discovered in this method. Re-analysis was performed after the outlier studies' removal to test the robustness of the results. Egger's test [15] and Duval and Tweedie's trim-and-fill method [16] were used to quantitatively assess the publication bias, and contour-enhanced funnel plots were built to visualize it.

### 2.8. Certainty Assessment

The Grading of Recommendations, Assessment, Development, and Evaluation (GRADE) ranking through five analysis criteria was used to evaluate the level of the evidence (risk of bias, inconsistency, indirect evidence, imprecision, and publication bias). As a result, the degree of certainty in the evidence was rated as high, moderate, low, or extremely low [17].

## 3. Results

### 3.1. Study Selection

**Figure 1:**
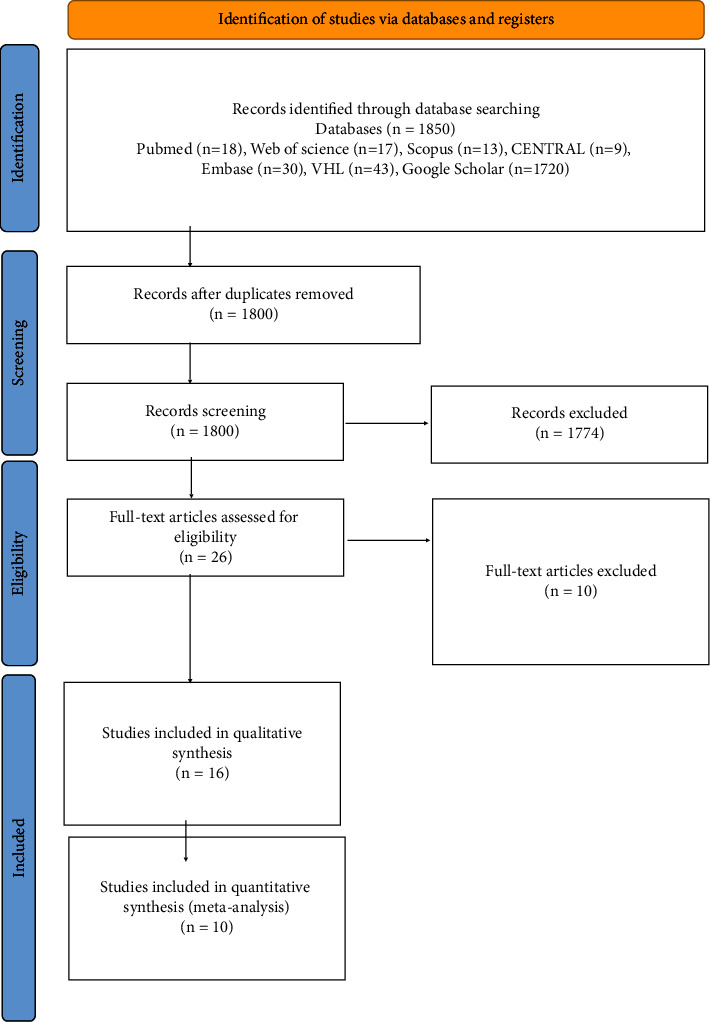
PRISMA flow diagram of the literature search.

1800 publications were found following a thorough search and the removal of duplicate studies. 26 studies were retained and evaluated for full-text evaluation after titles and abstracts were filtered according to the eligibility criteria. Hence, ten studies were excluded, and finally, sixteen RCTs [18–30] were assessed for qualitative synthesis and ten for meta-analysis [18, 21, 22, 25–29] (Figure 1).

### 3.2. Study Characteristics

#### 3.2.1. Review of the Included Studies

The main characteristics of the included studies are presented in Table 1. The papers compared the use of probiotics (experimental group) in any form with chlorhexidine mouthwash (comparator). Six studies had other experimental groups [18, 19, 21, 26]. Eleven studies had control groups using saline, mint water, distilled water, or regular oral hygiene measures [20, 22, 23, 25–30, 33]. The studies were conducted between 2010 and 2021.829 patients were evaluated. The number of participants ranged from 15 to 90, divided into experimental and comparator groups, with a mean of 51.8 participants. The included studies assessed whether daily oral administration of probiotics could influence the inflammatory response and plaque accumulation. Thirteen studies evaluated PI and GI [18–25, 27, 30, 33]. Three papers evaluated only PI [26, 28, 30]. Five papers also evaluated OHI-S [18, 22, 23, 30].

#### 3.2.2. Risk of Bias within Studies

**Figure 2:**
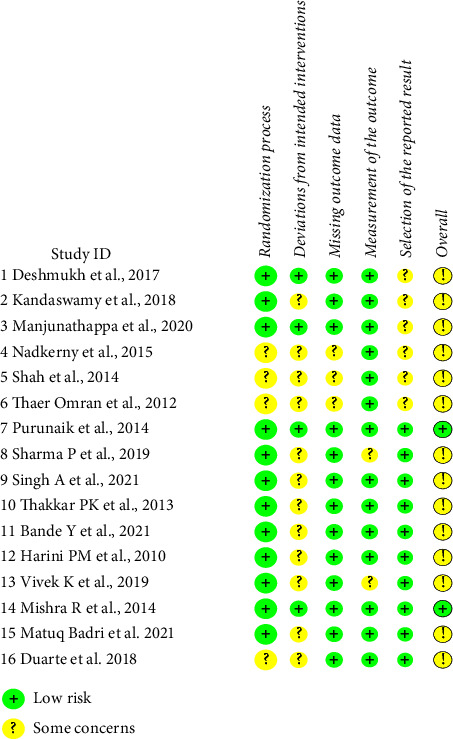
Summary of the risk-of-bias assessment.

The risk of bias within the included studies is presented in Figure 2. The majority of the included RCTs showed a moderate risk of bias and quality due to deviations from intended interventions [18–25, 27–30, 33]. On the other hand, two showed a low risk of bias [26, 33].

#### 3.2.3. Publication Bias Assessment

**Figure 3:**
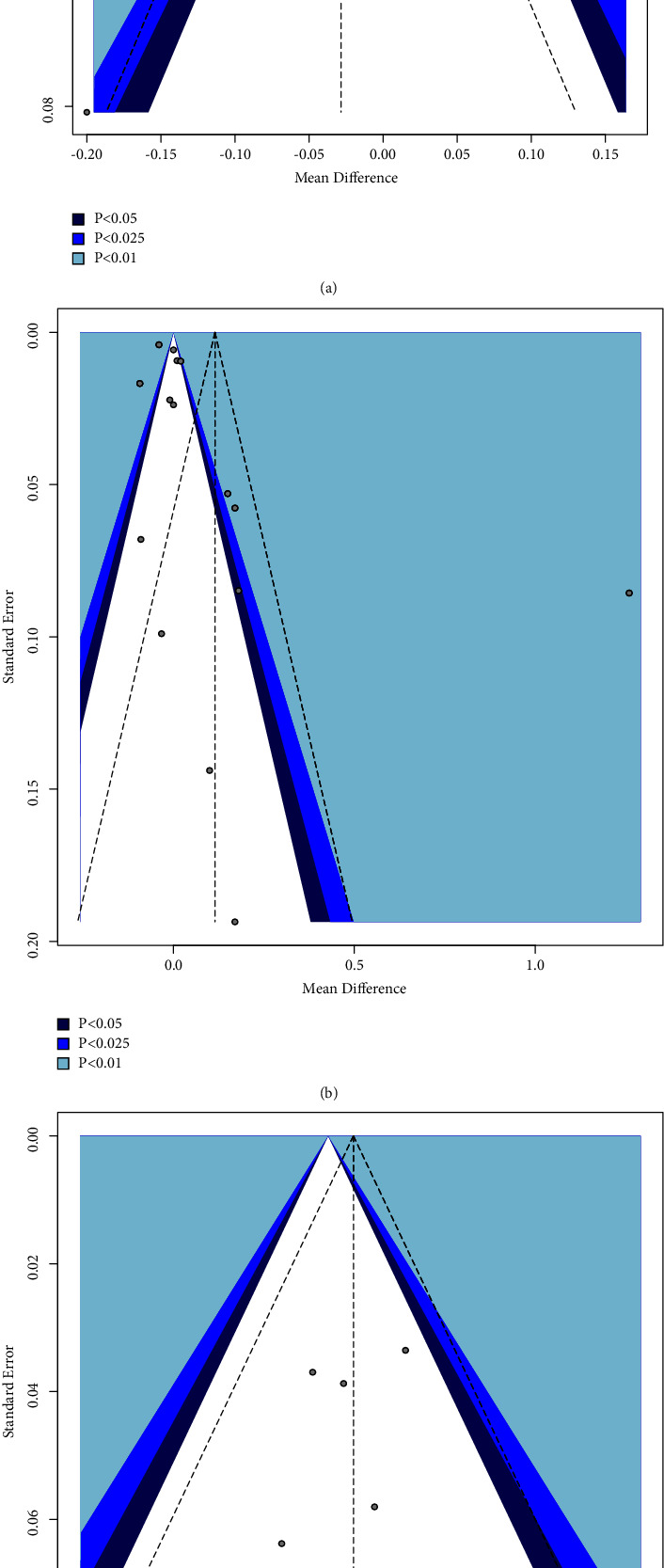
Contour-enhanced funnel plots visualizing publication bias. (a) GI outcome; (b) PI outcome; (c) OHI-S outcome. The vertical dashed line in the middle of the funnel shows the average effect size.

The results of the publication bias assessment are presented in Table 2 and Figure 3. Based on the quantitative tests, publication bias was not proved for all outcomes. However, according to the asymmetric pattern of funnel plots, publication bias was suspected to some extent.

## 4. Results of Meta-Analysis

### 4.1. Gingival Index

**Figure 4:**
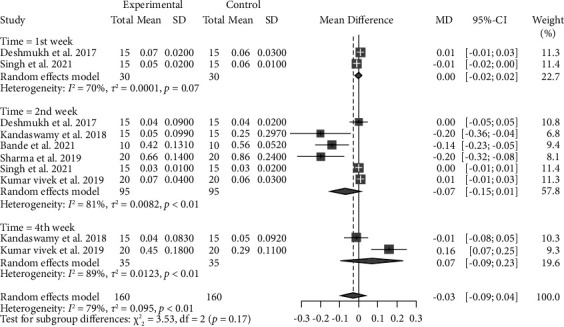
Forest plot for the gingival index (experimental: probiotics and control: chlorhexidine).

In terms of the gingival index, no statistically significant difference was observed between CHX and probiotics in all time intervals (WMD −0.03, 95% CI: −0.09∼0.04, *P* = 0.3885) with a very low level of evidence (Figure 4 and Supplementary Table 2). After removing outlier studies, similar to the previous analysis, no significant difference was observed in all time points (WMD −0.05, 95% CI: −0.11∼0.01, *P* = 0.0971) with a very low level of evidence (Supplementary Figures 1 and 2 and Supplementary Table 2).

#### 4.1.1. Plaque Index

**Figure 5:**
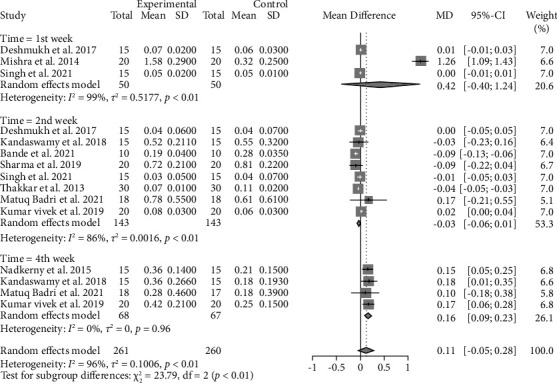
Forest plot for the plaque index (experimental: probiotics and control: chlorhexidine).

Similar to GI, no statistically significant difference was observed between CHX and probiotics regarding PI (WMD 0.11, 95% CI: −0.05∼0.28, *P* = 0.1726) with a very low level of evidence (Figure 5 and Supplementary Table 2). On the contrary, a statistically significant difference was shown in the fourth week after using mouthwashes (WMD 0.16, 95% CI: −0.05∼0.28), which favored chlorhexidine, with a moderate level of evidence (Figure 5 and Supplementary Table 2). Moreover, after removing outliers, using sensitivity analysis, no statistically significant difference was observed between CHX and probiotics regarding PI (WMD 0.01, 95% CI: −0.03∼0.05, *P* = 0.5272) with a very low level of evidence (Supplementary Figures 3 and 4 and Supplementary Table 2).

#### 4.1.2. Oral Hygiene Index-Simplified (OHI-S)

**Figure 6:**
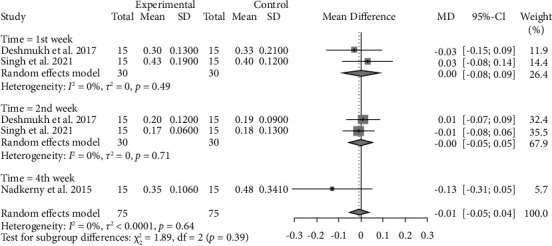
Forest plot for the oral hygiene index (experimental: probiotics and control: chlorhexidine).

No statistically significant difference was observed between CHX and probiotics in all time intervals regarding OHI-S after performing sensitivity analysis (WMD −0.01, 95% CI: −0.05∼0.04, *P* = 0.7508) with a moderate level of evidence (Figure 6 and Supplementary Table 2).

## 5. Discussion

Concerning chlorhexidine's long-term side effects, researchers have been looking for an alternative agent to improve the periodontal status and manage periodontal diseases. Thus, probiotics have been the subject of many clinical trials to prove their efficacy in reducing plaque accumulation and gingival inflammation [8].

The current systematic review investigated randomized clinical trials to assess the effectiveness of probiotics as an alternative to chlorhexidine for the management of periodontal status. All the included papers used periodontal clinical parameters (plaque index and/or gingival index) to evaluate interventions in follow-ups with different mouthwashes and probiotic lozenges [18–30, 33] [29–30, 33].

Ten studies included in the meta-analysis used the Silness–Loe plaque index (1964) [18, 21, 22, 25–30], nine papers used the Loe–Silness gingival index (1963) [18, 21, 22, 25–28, 30], and five clinical trials used oral hygiene index-simplified (Green and Vermillion) [18, 22, 23, 30]. The data were numerically similar and were measured using the same techniques; hence, the weighted mean difference (WMD) was used to pool effect sizes.

After pooling effect sizes, no statistically significant difference was observed between probiotics and chlorhexidine mouthwash within 4 weeks of follow-up. There was no significant statistical difference found in the gingival index. This finding demonstrates that probiotics have been shown to improve inflammatory response. It is well known that probiotics contain beneficial commensals, which operate as a natural barrier against bacteria [5–7, 34]. Probiotics lessen bacterial adhesion to tooth surfaces, which may prevent microbial growth and proliferation as well as the development of the intercellular plaque matrix. It demonstrates the value of utilizing probiotics by altering the biochemistry of plaque, preventing the production of cytotoxic products that alter the ecology of plaque, and preventing toxicities and antibiotic resistance [5–7, 34].

However, these findings were based on very low to moderate certainty of the evidence. The analyses showed very low levels of certain evidence for the gingival index and plaque index outcomes due to a large amount of heterogeneity and the lack of precision. Rücker et al. have proposed three sources of heterogeneity in meta-analyses including clinical heterogeneity (such as differences between sample characteristics), statistical heterogeneity, and other sources of heterogeneity (such as design-related heterogeneity) [35]. Studies that used various probiotic formulations and concentrations of chlorhexidine may have contributed to the observed heterogeneity. On the other hand, blindness varied among the included studies.

Age, weight, and adherence to oral hygiene advice are a few variables that may have an impact on periodontal health. Moreover, previous research has shown that obesity serves as a potentiator of the periodontal condition because of the elevation of inflammatory cytokines [36]. These characteristics might have a variety of effects on a heterogeneous sample, making it more challenging for the systematic review's external validity.

Also, the presence of publication bias was suspected based on the asymmetrical pattern of the funnel plots for outcomes. However, publication bias was not proved using statistical tests. It is crucial, however, not to jump to conclusions and interpret the funnel plot cautiously. In fact, publication bias is just one of many possible reasons for funnel plot asymmetry. Small studies with extremely large effect sizes, between-study heterogeneity, and different study designs can lead to funnel plot asymmetry [37]. It is a common finding that low-quality studies tend to show larger effect sizes because there is a higher risk of bias. Large studies require more investment, so, likely, their methodology will also be more rigorous [37].

As a result, these findings should be interpreted cautiously. To standardize comparisons, we advise that future randomized clinical studies be conducted using the same probiotic composition and chlorhexidine concentration.

We advise conducting more longitudinal studies and microbiological testing before prescribing probiotics as an antiseptic and antibacterial agent. Due to the inclusion of low to moderate-quality primary papers, this review was constrained. Furthermore, the probiotic and chlorhexidine comparison in these clinical trials lacked standardization. They employed several types of probiotics, chlorhexidine formulations, and concentrations. The aforementioned issues should be considered in future studies.

## 6. Conclusion

Probiotics are an alternate option for enhancing periodontal health. It might also serve as a substitute for chlorhexidine mouthwash to avoid any potential negative effects. To determine the ideal doses for clinical implications, additional high-quality research with strict methods should be conducted.

## Figures and Tables

**Table 1:** Characteristics of the included studies.

First author/year	Design	*N* Age range (years)	Experimental	Comparator	Clinical parameters	Follow-up (days)	Main findings
[18]	SB-RCT	45 18–21	Group A: HiOra regular mouthwash (The Himalaya Drug Company) Group B: Darolac sachets (Aristo Pharmaceuticals Pvt. Ltd.)	Chlorhexidine gluconate 0.2% c (ICPA Health Products Ltd.)	OHI-S PI GI	0–7–14	Three types of mouthwashes (chlorhexidine, herbal, and probiotic) were equally effective in improving periodontal health
[21]	SB-RCT	45 10–12	Group A: Bifilac sachets (Allianz Biosciences Pvt Ltd., Puducherry, India) containing the probiotic powder Group B: sesame oil (Idhayam group of companies, Virudhunagar, India)	Chlorhexidine mouthwash (Rexidin, Warren-Indoco Remedies Ltd., Mumbai, India	PI GI	0–15–30	The use of probiotic mouthwash, chlorhexidine mouthwash, and sesame oil pulling therapy was equally effective in the reduction of plaque and the improvement of the gingival status
[19]	TB-RCT	60 12–15	Group A: tulsi (*Ocimum sanctum)* extract mouth rinse Group B: probiotic mouth rinse (Sporlac)	Chlorhexidine mouth rinse 0.2% (Clohex, concentration 0.2%)	PI GI	0–21	All mouth rinses showed a significant reduction in plaque and gingivitis. Probiotic mouth rinse was more effective in reducing gingivitis, followed by chlorhexidine and tulsi mouth rinses
[22]	SB-RCT	45 20–30	Group A: probiotic mouth rinse (Sporlac Plus® (Sanzyme Ltd. India) + distilled water) Group B: control (saline)	Chlorhexidine mouthwash 0.02% (Hexidine® (ICPA))	OHI-S PI GI	0–14–28	Probiotic mouth rinse was effective in reducing plaque accumulation and gingival inflammation
[31]	DB-RCT	40 20–30	Group A: probiotic Group B: fluoride Group C: control	Chlorhexidine	PI GI	0–28	Probiotic mouth rinse was effective in reducing plaque accumulation and gingival inflammation in 6–10-year-old children
[23]	SB-RCT	15 2–300	Group A: probiotic (BreathActive Cleanition, Switzerland) Group B: control (distilled water)	Chlorhexidine 0.2% (Parodontax® Extra, GSK, Germany)	OHI-S PI GI	OHI-S: 6–12 GI: 6–12 PI: 0–30	Probiotics demonstrated better treatment results when compared to mechanical treatment alone and were comparable to chlorhexidine
[20]	DB-RCT	45 6–8	Group A: control group (mint water) Group B: probiotic	Chlorhexidine	PI GI	0–14	Probiotic mouth rinse was effective in reducing plaque accumulation and gingival inflammation
[27]	SB-RCT	60 6–9	Group A: control (distilled water) Group B: probiotic (Darolac, Aristo Pharmaceuticals, India)	Chlorhexidine 0.02% (Hexidine)	PI GI	0–3–14	Extensive research was recommended to determine the strain of probiotics, the appropriate vehicle, and the amount of probiotic administration so that they can be effectively used and recommended by clinicians as an effective tool in plaque control and maintaining periodontal health
[26]	DB-RCT	60 6–14	Group A: probiotic mint tablet mixed with water (Evora Plustm, Florida, USA) Group B: herbal oral rinse (Herboral, M-Tech Innovations Ltd., Pune, India)	Chlorhexidine 0.2% (Hexidine, IPCA Health Products Ltd., India)	PI	0–7	Herbal mouth rinse showed an equal antimicrobial effect compared to chlorhexidine digluconate 0.2% in reducing plaque accumulation, whereas probiotic mouth rinse showed less effectiveness
[32]	SB-RCT	45 20–30	Group A: green tea (Lipton green tea bags) Group B: probiotic (Darolac, Aristo Pharmaceuticals, India)	Chlorhexidine	OHI-S PI GI	0–7–14	Probiotics and green tea were effective in reducing dental plaque
[28]	SB-RCT	90 13–15	Group A: placebo mouth rinse (colored distilled water) Group B: probiotic mouth rinse (Darolac)	Chlorhexidine 0.12% (Carix® KIN S.A. Laboratory, Spain)	PI	0–14–36	Probiotic mouth rinse and chlorhexidine mouth rinse caused significant inhibition of dental plaque accumulation, but probiotic mouth rinse was found to be more effective for inhibition of dental plaque accumulation after 14 days of intervention and 3 weeks after discontinuation of intervention
[25]	DB-RCT	30 6–8	Group A: control (mint water) Group B: probiotic (Darolac, Lallemand Health Solutions, India)	Chlorhexidine	PI GI	0–14	Probiotic mouth rinse showed a potential therapeutic effect and was an effective and safe alternative to chlorhexidine mouthwash
[33]	DB-RCT	90 15-16	Group A: probiotic (Darolac, Aristo Pharmaceuticals, India) Group B: placebo (distilled water)	Chlorhexidine 0.2% (Clohex)	PI GI	0–14	There was a statistically significant difference between chlorhexidine and probiotic mouth rinse
[30]	SB-RCT	60 25–35	Group A: probiotic (Darolac sachets) Group B: control (saline)	Chlorhexidine 0.2% (Rexidin, Warren-Indoco Remedies Ltd., Mumbai, India)	PI GI OHI-S	0–14–28	Probiotic mouth rinse was effective in reducing plaque accumulation and gingival inflammation
[29]	SB-RCT	54 8–12	Group A: probiotic lozenge (BioGaia Prodentis) Group B: control group (only instructed to follow regular oral hygiene measures)	Chlorhexidine 0.05% (Clorasept)	PI	0–15–30	Probiotic lozenges and chlorhexidine significantly reduced plaque accumulation
[24]	SB-RCT	45 25–35	Group A: control group (mint water) Group B: probiotic dairy product group	Chlorhexidine 0.02%	PI GI	0–14	There was a significant difference in the mean PI and mean GI between the control, chlorhexidine, and probiotic groups after 14 days. However, there were no significant differences in terms of plaque accumulation between the probiotic and chlorhexidine groups on day 14

SB-RCT: single-blinded randomized clinical trial; TB-RCT: triple-blinded randomized clinical trial; DB-RCT: double-blinded randomized clinical trial; OHI-S: oral hygiene index-simplified; PI: plaque index; GI: gingival index.

**Table 2:** Results of the publication bias.

Outcomes	Funnel plot	Eggers' test (*P* value)	Trim-fillmethod (*P* value)
GI	Asymmetric	0.680	0.410
PI	Asymmetric	0.079	0.858
OHI-S	Asymmetric	0.751	0.390

OHI-S: oral hygiene index-simplified; PI: plaque index; GI: gingival index.

## Data Availability

Data are available from the corresponding author upon request.

## Supplementary Materials

Supplementary Materials
*Supplementary Figure 1.* Detecting outlier studies for the gingival index. *Supplementary Figure 2.*Meta-analysis after removing outlier studies for the gingival index. *Supplementary Figure 3.* Detecting outlier studies for the plaque index. *Supplementary Figure 4.*Meta-analysis after removing outlier studies for the plaque index. *Supplementary Table 1.* Databases and search strategy. *Supplementary Table 2.* GRADE certainty of the evidence.
