# Supplementary material for: Efficacy of Probiotics Compared to Chlorhexidine Mouthwash in Improving Periodontal Status: A Systematic Review and Meta-Analysis
Source: Int J Dent. 2023 Jan 23;2023:4013004. doi: 10.1155/2023/4013004 (PMC9886484; doi:10.1155/2023/4013004)
Supplement: Supplementary Materials — Supplementary Figure 1. Detecting outlier studies for the gingival index. Supplementary Figure 2.Meta-analysis after removing outlier studies for the gingival index. Supplementary Figure 3. Detecting outlier studies for the plaque index. Supplementary Figure 4.Meta-analysis after removing outlier studies for the plaque index. Supplementary Table 1. Databases and search strategy. Supplementary Table 2. GRADE certainty of the evidence. [file 4013004.f1.zip › Supplementary Table 1 (2).docx]

| Medline via PubMed: 18 (11/12/2021) |
| --- |
| ((probiotic*) AND (chlorhexidine)) AND (periodontitis OR periodont* OR gingivitis) |
| Web of Science: 17 (11/12/2021) |
| TS=(probiotic*) AND TS=(chlorhexidine*) AND TS=(periodont*) |
| Scopus: 13 (11/12/2021) |
| TITLE-ABS-KEY (probiotics) AND TITLE-ABS- KEY ("chlorhexidine”) AND TITLE-ABS- KEY ( "periodontal diseases" OR "gingivitis" OR "periodontitis") |
| VHL: 43 (11/12/2021) |
| (probiotic) AND (chlorhexidine) AND (periodontitis) OR (“periodontal disease”) OR (gingivitis) |
| Embase: 30 (11/12/2021) |
| (‘probiotic’/exp OR probiotic) AND (‘chlorhexidine/exp OR chlorhexidine) AND (‘periodontitis’/exp OR periodontitis OR ‘periodontal disease’/exp OR periodontal disease OR ‘gingivitis’/exp OR gingivitis) |
| CENTRAL (via Cochrane Library): 9 (11/12/2021) |
| (probiotic*) AND (chlorhexidine) AND (periodontitis OR ‘periodontal disease’ OR gingivitis) |
| Google Scholar: 1720 (11/12/2021) |
| (probiotic*) AND (chlorhexidine) AND (periodontitis OR ‘periodontal disease*` OR gingivitis) |
