# Supplementary material for: Efficacy of Probiotics Compared to Chlorhexidine Mouthwash in Improving Periodontal Status: A Systematic Review and Meta-Analysis
Source: Int J Dent. 2023 Jan 23;2023:4013004. doi: 10.1155/2023/4013004 (PMC9886484; doi:10.1155/2023/4013004)
Supplement: Supplementary Materials — Supplementary Figure 1. Detecting outlier studies for the gingival index. Supplementary Figure 2.Meta-analysis after removing outlier studies for the gingival index. Supplementary Figure 3. Detecting outlier studies for the plaque index. Supplementary Figure 4.Meta-analysis after removing outlier studies for the plaque index. Supplementary Table 1. Databases and search strategy. Supplementary Table 2. GRADE certainty of the evidence. [file 4013004.f1.zip › Supplementary table 2 (1).pdf]

Supplementary File- Certain of evidence by GRADE analyze

Question: [Probiotic] compared to [Chlorhexidine] for [problem periodontal]

Certainty assessment							№ of patients		Effect		Certainty	Importance
№ of studies	Study design	Risk of bias	Inconsistency	Indirectness	Imprecision	Other considerations	[Probiotic]	[Chlorhexidine]	Relative (95% CI)	Absolute (95% CI)		
**Gingival index (follow-up: range 1 weeks to 4 weeks)**												
10	randomised trials	not serious	very serious[a]	not serious	serious[b]	none	160	160	-	MD **0.03 lower** (0.09 lower to 0.04 higher)	⊕◯◯◯ Very low	CRITICAL
**Gingival index outliers removal (follow-up: range 1 weeks to 4 weeks)**												
8	randomised trials	not serious	very serious[a]	not serious	serious[b]	none	120	120	-	**0** (0 to 0)	⊕◯◯◯ Very low	CRITICAL
**Paque index (follow-up: range 1 weeks to 4 weeks)**												
15	randomised trials	not serious	very serious[a]	not serious	serious[b]	none	261	260	-	MD **0.11 higher** (0.05 lower to 0.28 higher)	⊕◯◯◯ Very low	CRITICAL
**Plaque index outliers removal (follow-up: range 1 weeks to 4 weeks)**												
14	randomised trials	not serious	very serious[a]	not serious	serious[b]	none	241	240	-	**0** (0 to 0)	⊕◯◯◯ Very low	CRITICAL
**Oral Hygiene Index outliers removal (follow-up: range 1 weeks to 4 weeks)**												
5	randomised trials	not serious	not serious	not serious	serious[b]	none	75	75	-	MD **0.01 lower** (0.05 lower to 0.04 higher)	⊕⊕⊕◯ Moderate	CRITICAL
**Plaque index (follow-up: 4 weeks)**												
4	randomised trials	not serious	not serious	not serious	serious[a]	none	68	67	-	MD **0.16 higher** (0.09 higher to 0.23 higher)	⊕⊕⊕◯ Moderate	CRITICAL

CI: confidence interval; **MD:** mean difference

Explanations

a. There is considerable statistically significant heterogeneity (>75%)
b. Confidence interval crosses null value or includes values favoring either treatment
