# Supplementary figures and images for: Efficacy of Probiotics Compared to Chlorhexidine Mouthwash in Improving Periodontal Status: A Systematic Review and Meta-Analysis
Source: Int J Dent. 2023 Jan 23;2023:4013004. doi: 10.1155/2023/4013004 (PMC9886484; doi:10.1155/2023/4013004)

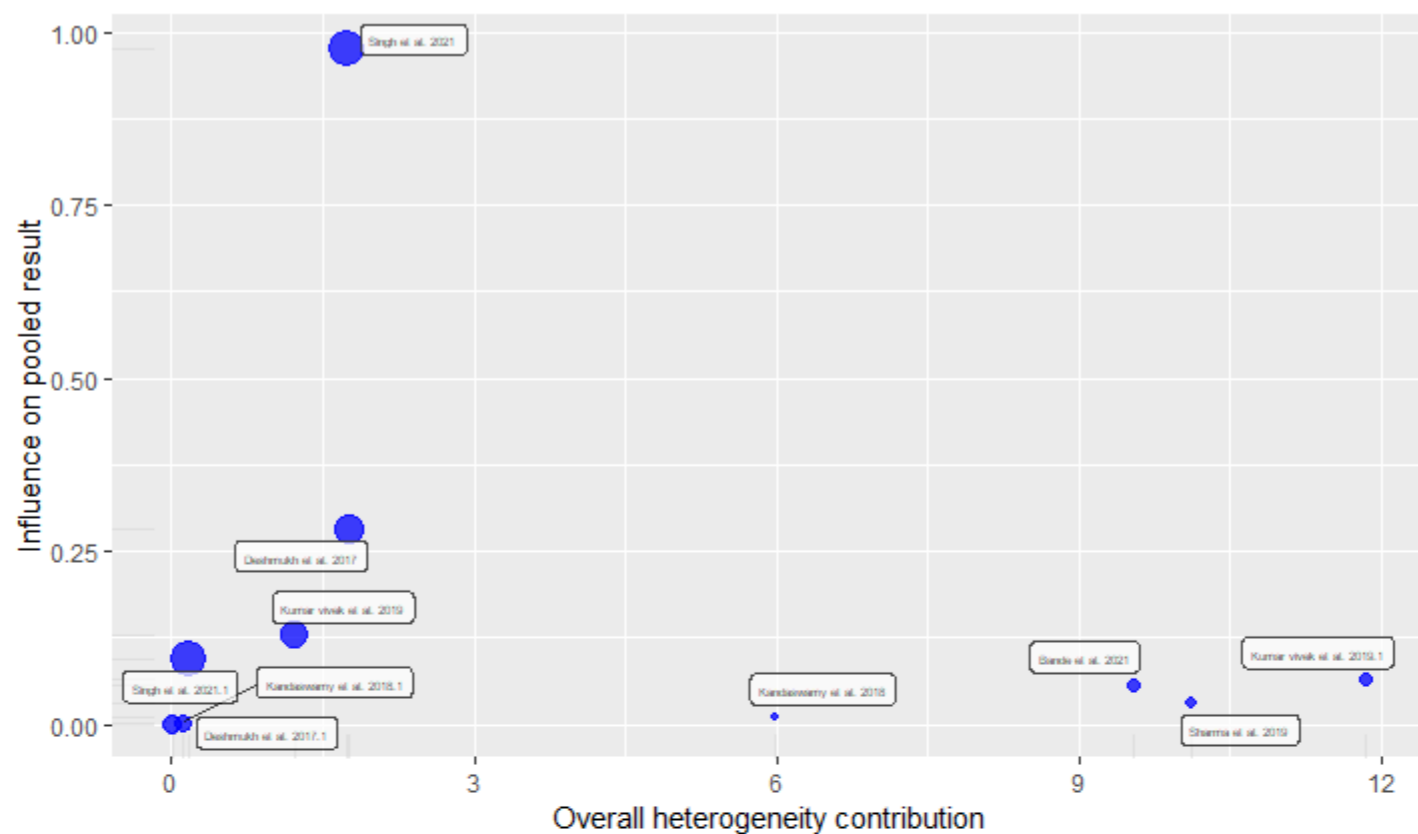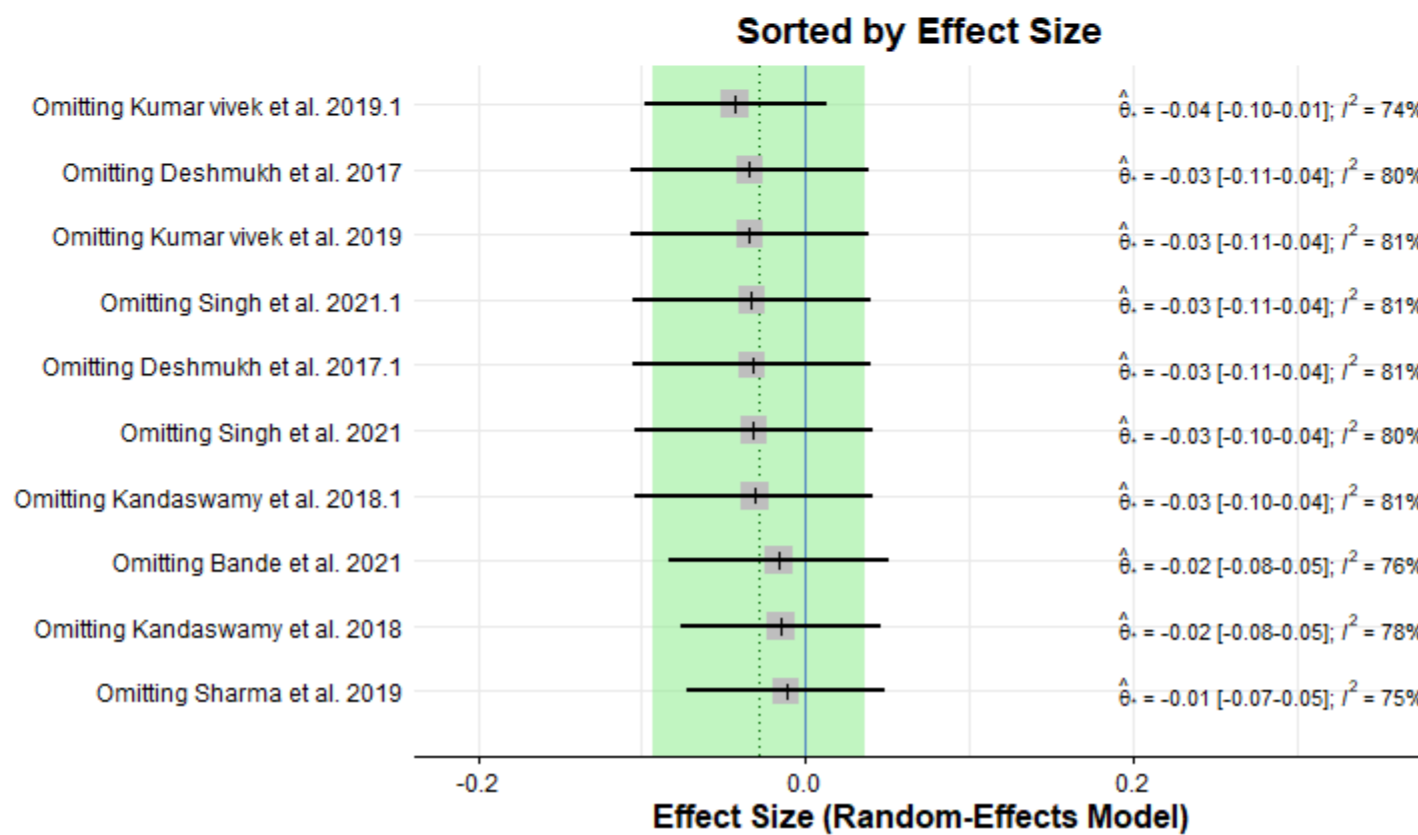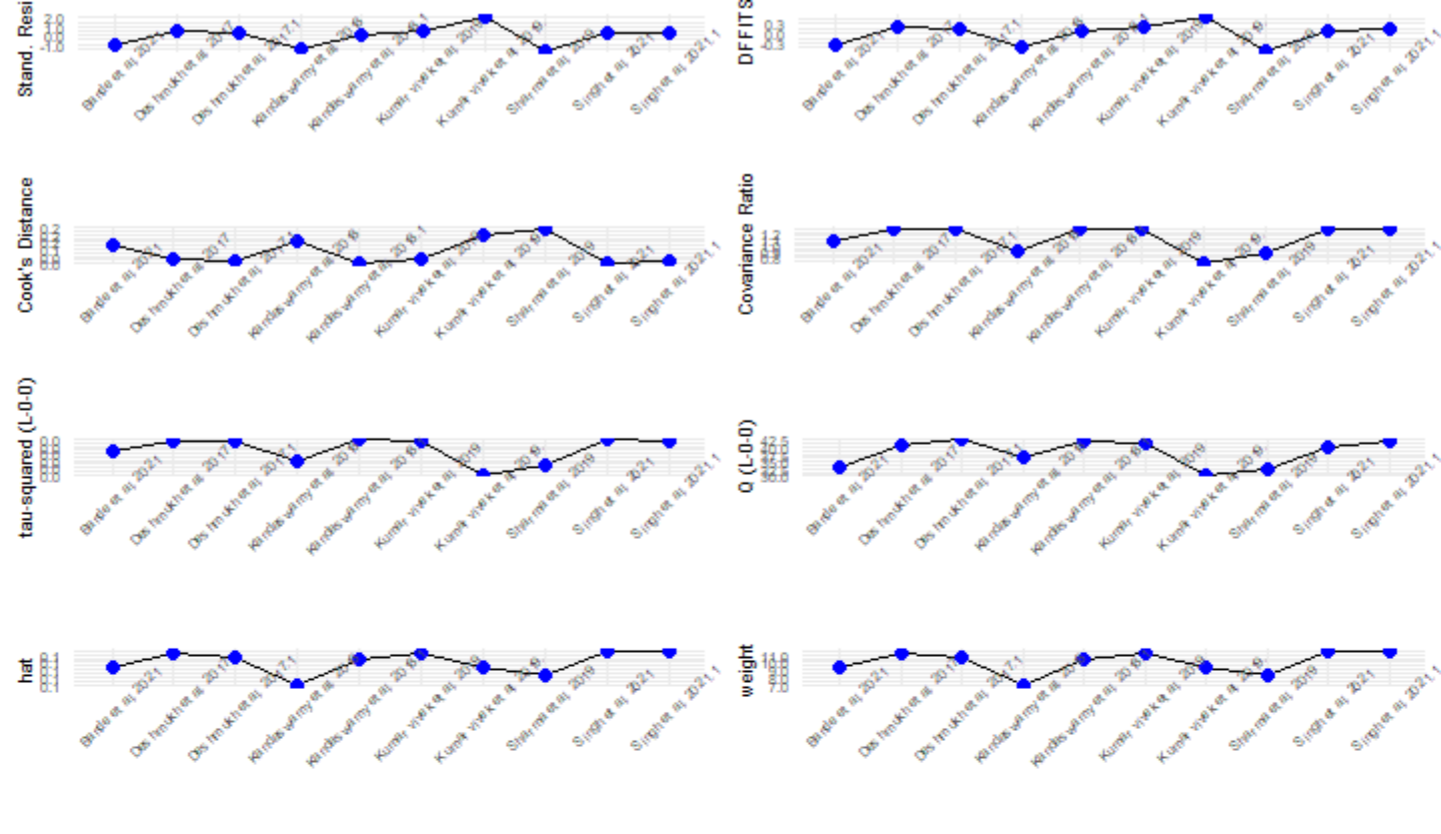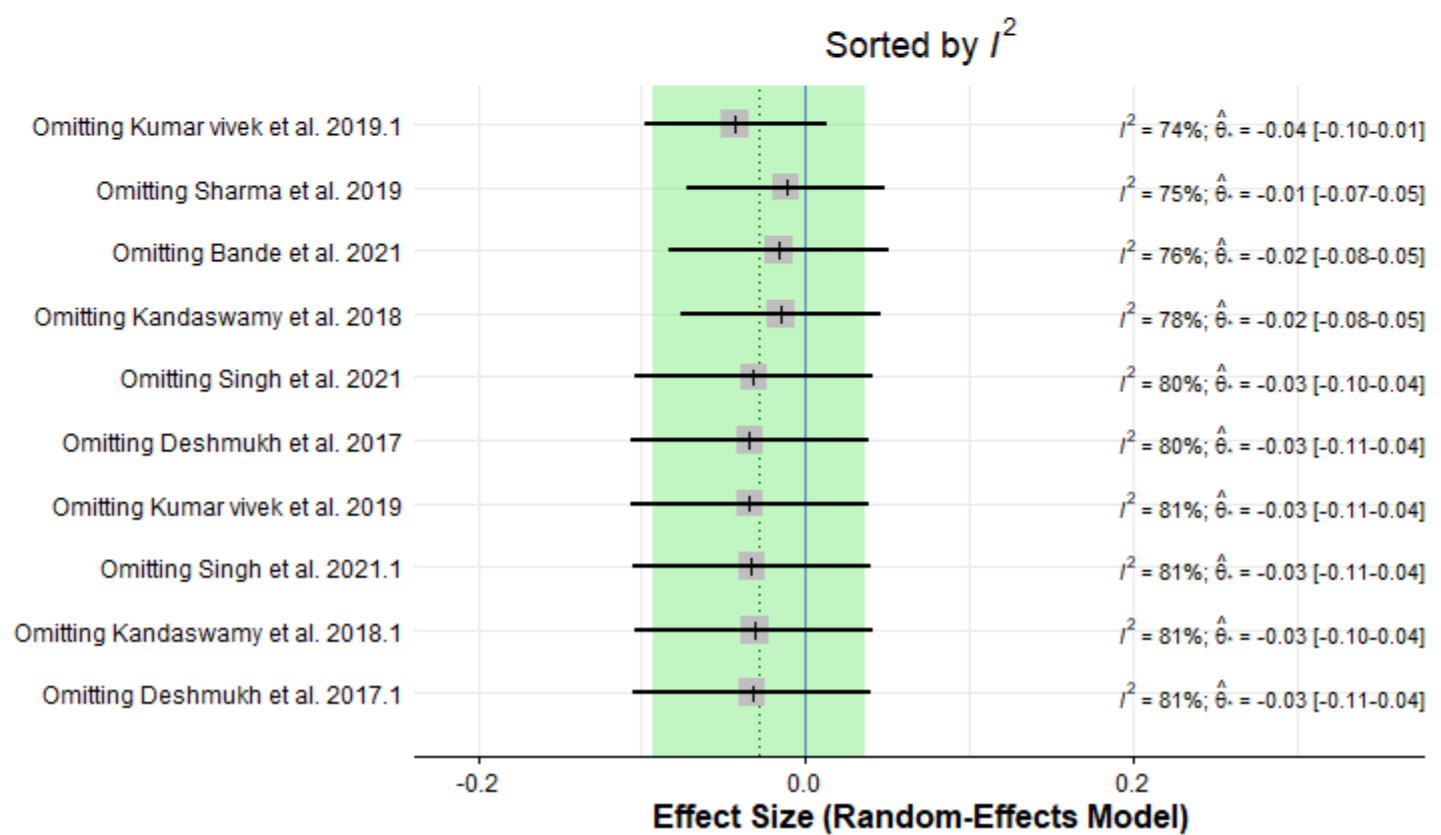

Supplement: Supplementary Materials — Supplementary Figure 1. Detecting outlier studies for the gingival index. Supplementary Figure 2.Meta-analysis after removing outlier studies for the gingival index. Supplementary Figure 3. Detecting outlier studies for the plaque index. Supplementary Figure 4.Meta-analysis after removing outlier studies for the plaque index. Supplementary Table 1. Databases and search strategy. Supplementary Table 2. GRADE certainty of the evidence. [file 4013004.f1.zip › Supplementary figure 1.pdf]

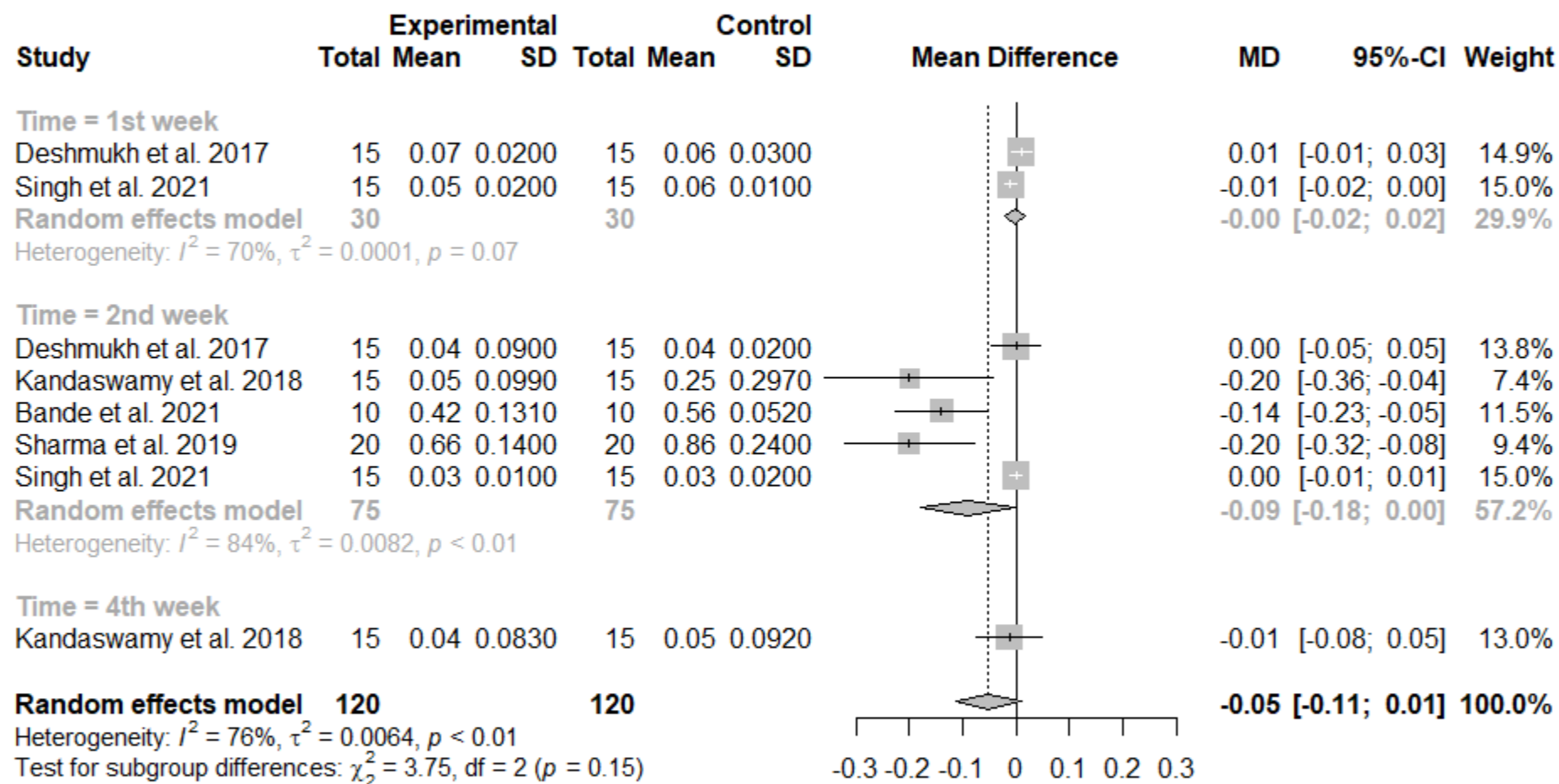

Supplement: Supplementary Materials — Supplementary Figure 1. Detecting outlier studies for the gingival index. Supplementary Figure 2.Meta-analysis after removing outlier studies for the gingival index. Supplementary Figure 3. Detecting outlier studies for the plaque index. Supplementary Figure 4.Meta-analysis after removing outlier studies for the plaque index. Supplementary Table 1. Databases and search strategy. Supplementary Table 2. GRADE certainty of the evidence. [file 4013004.f1.zip › Supplementary figure 2.pdf]

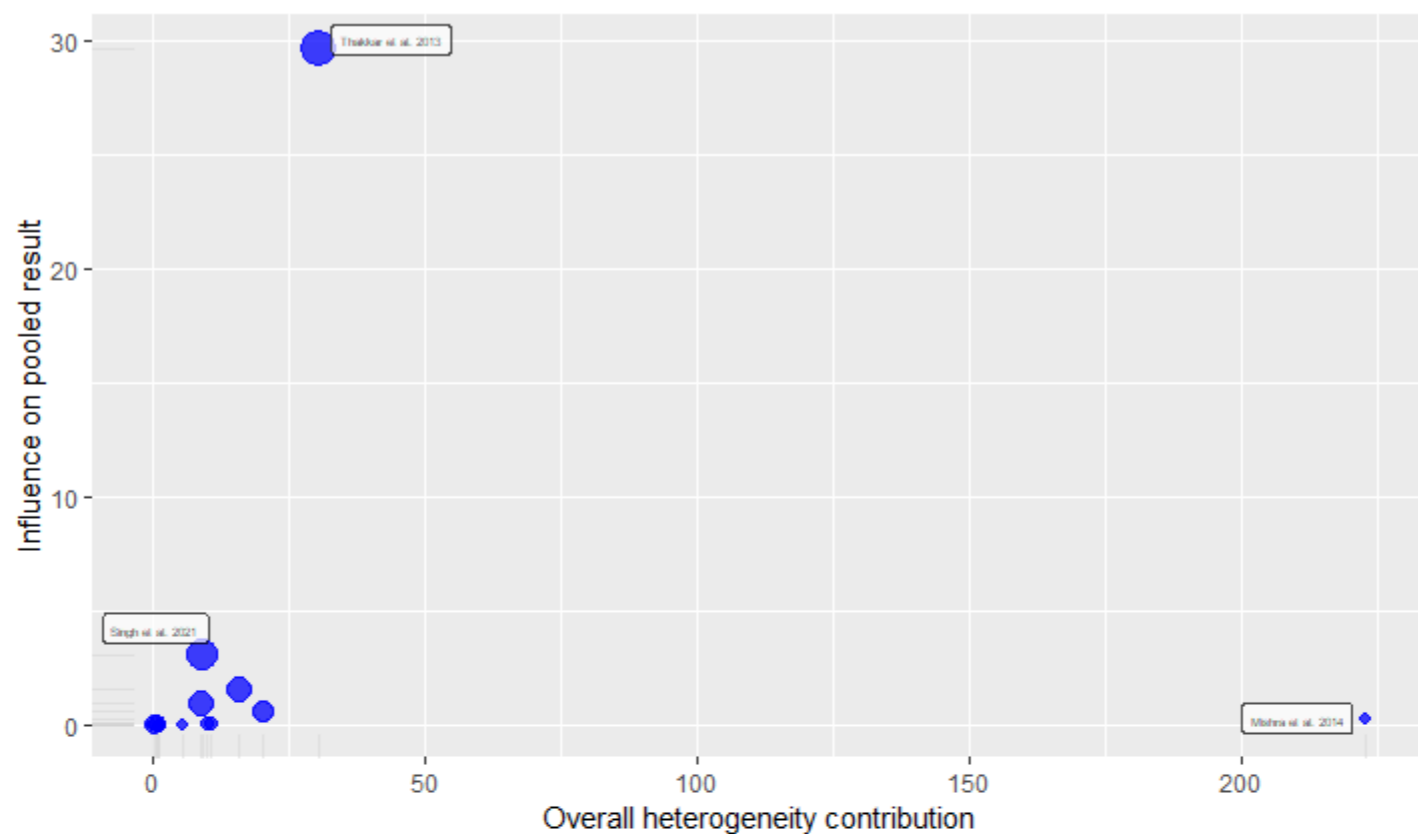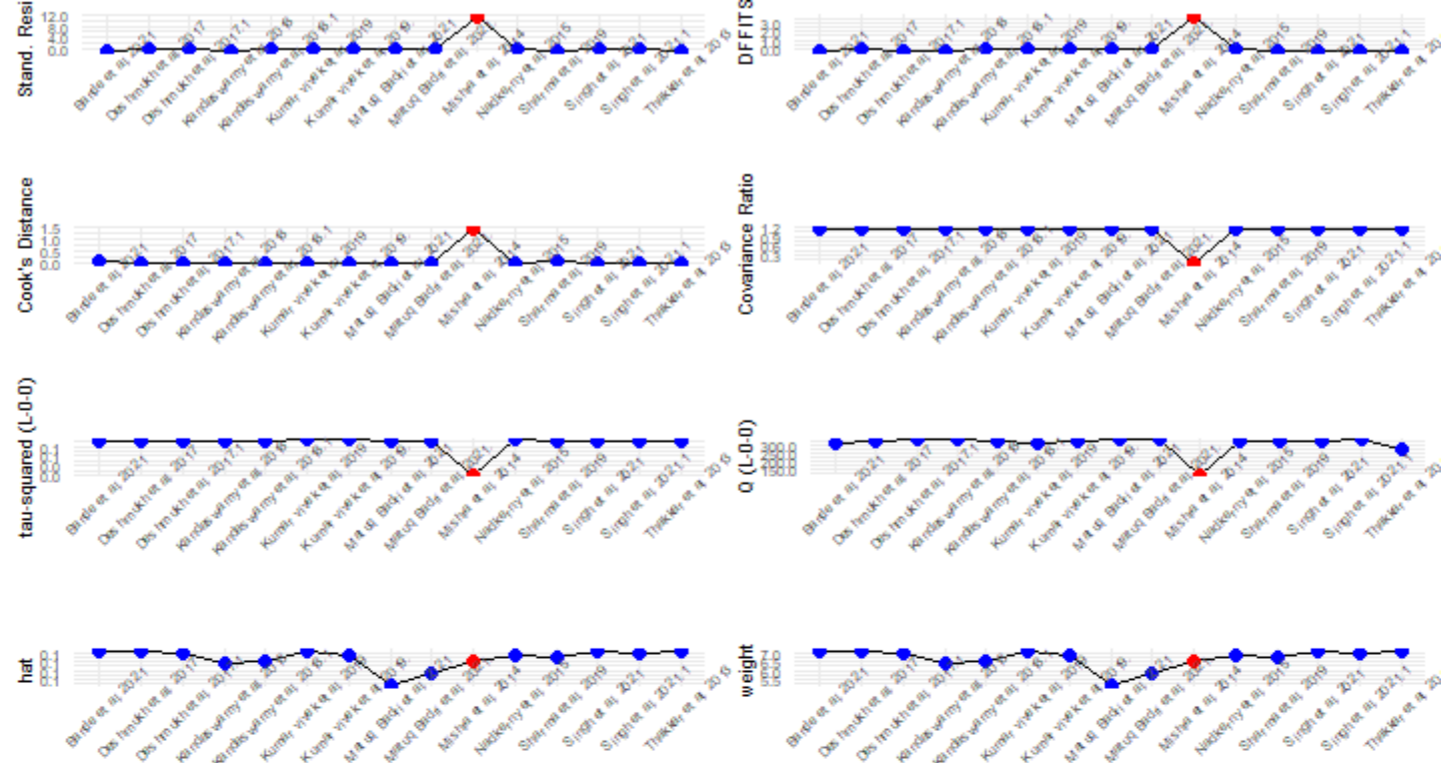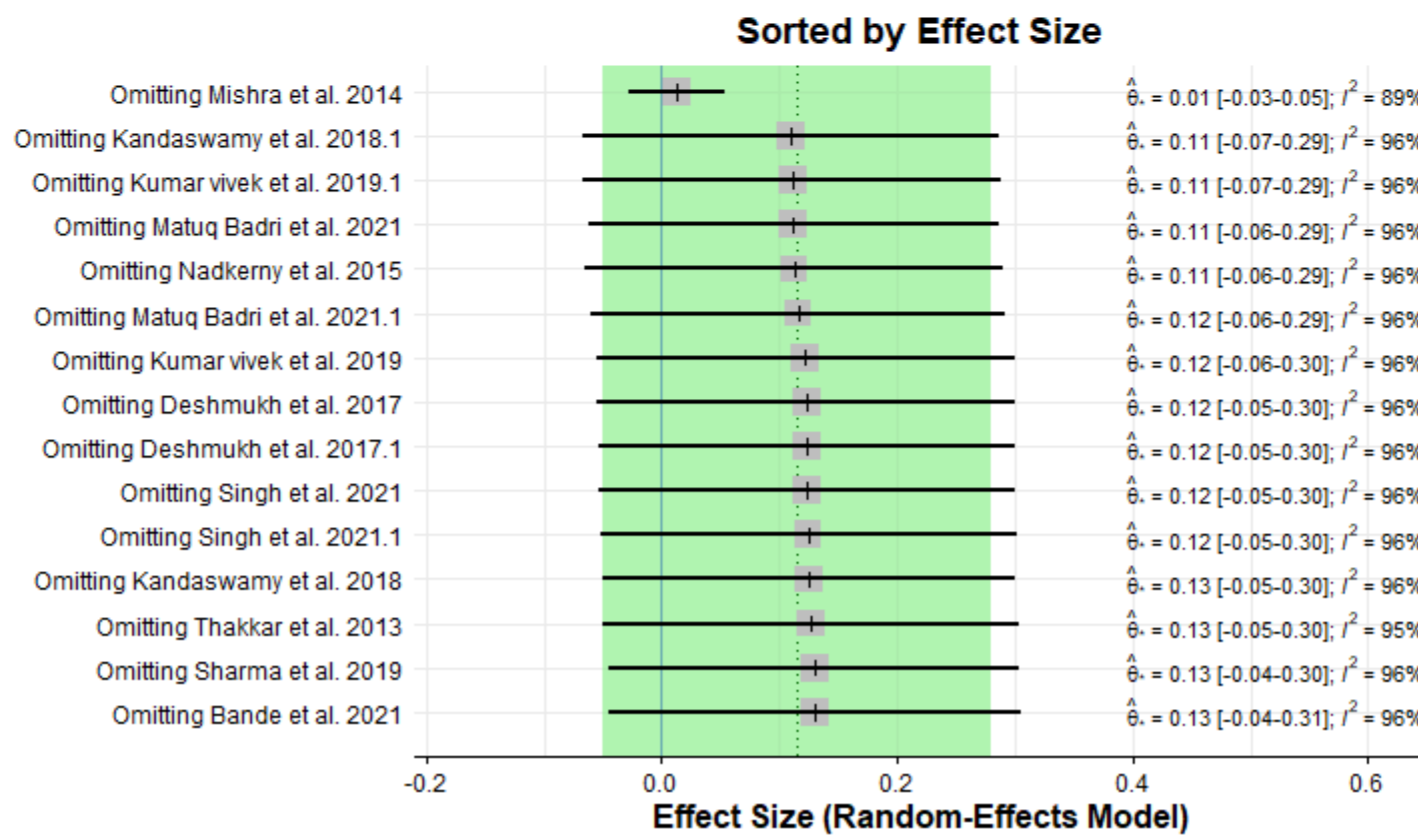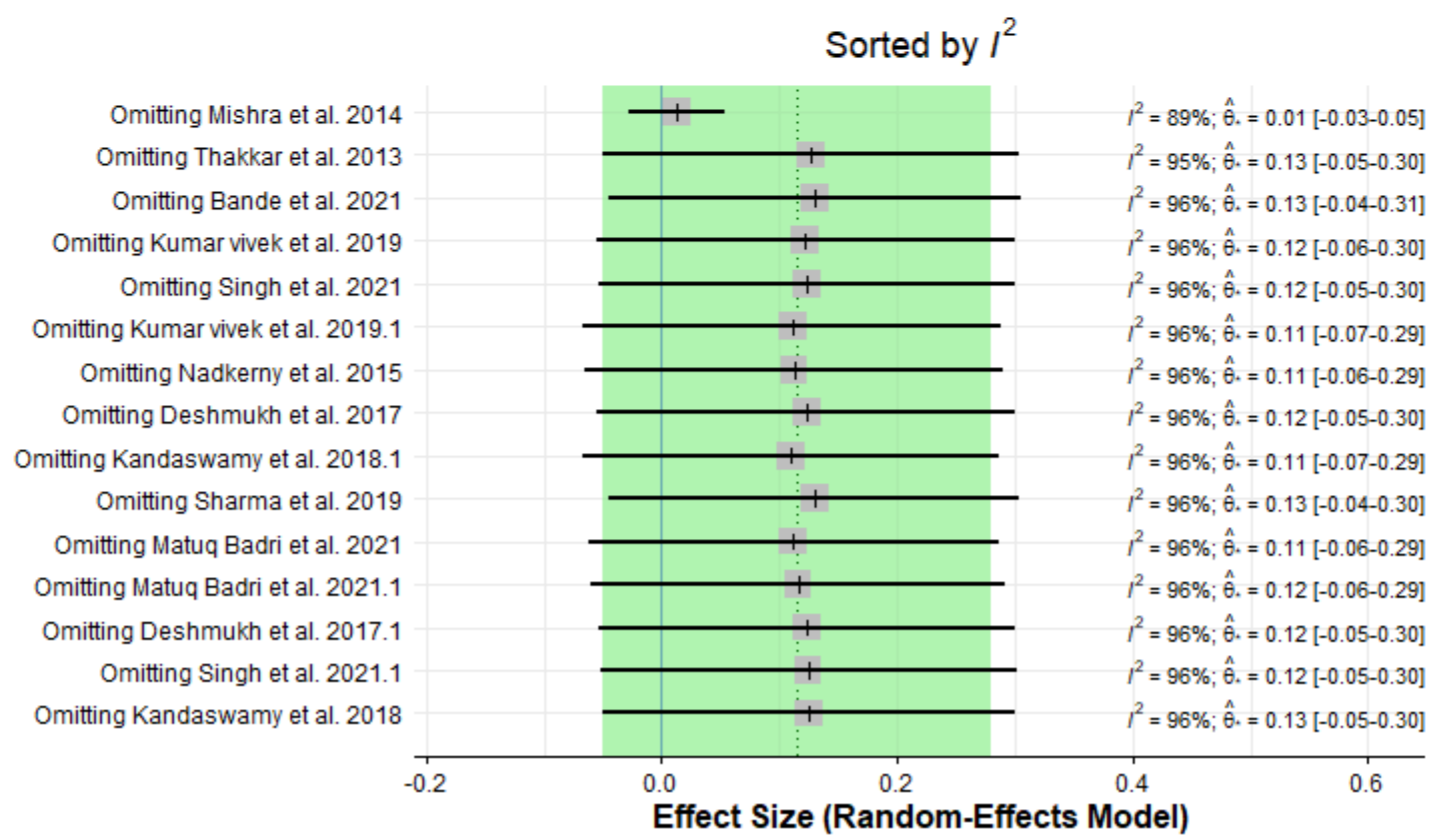

Supplement: Supplementary Materials — Supplementary Figure 1. Detecting outlier studies for the gingival index. Supplementary Figure 2.Meta-analysis after removing outlier studies for the gingival index. Supplementary Figure 3. Detecting outlier studies for the plaque index. Supplementary Figure 4.Meta-analysis after removing outlier studies for the plaque index. Supplementary Table 1. Databases and search strategy. Supplementary Table 2. GRADE certainty of the evidence. [file 4013004.f1.zip › Supplementary figure 3.pdf]

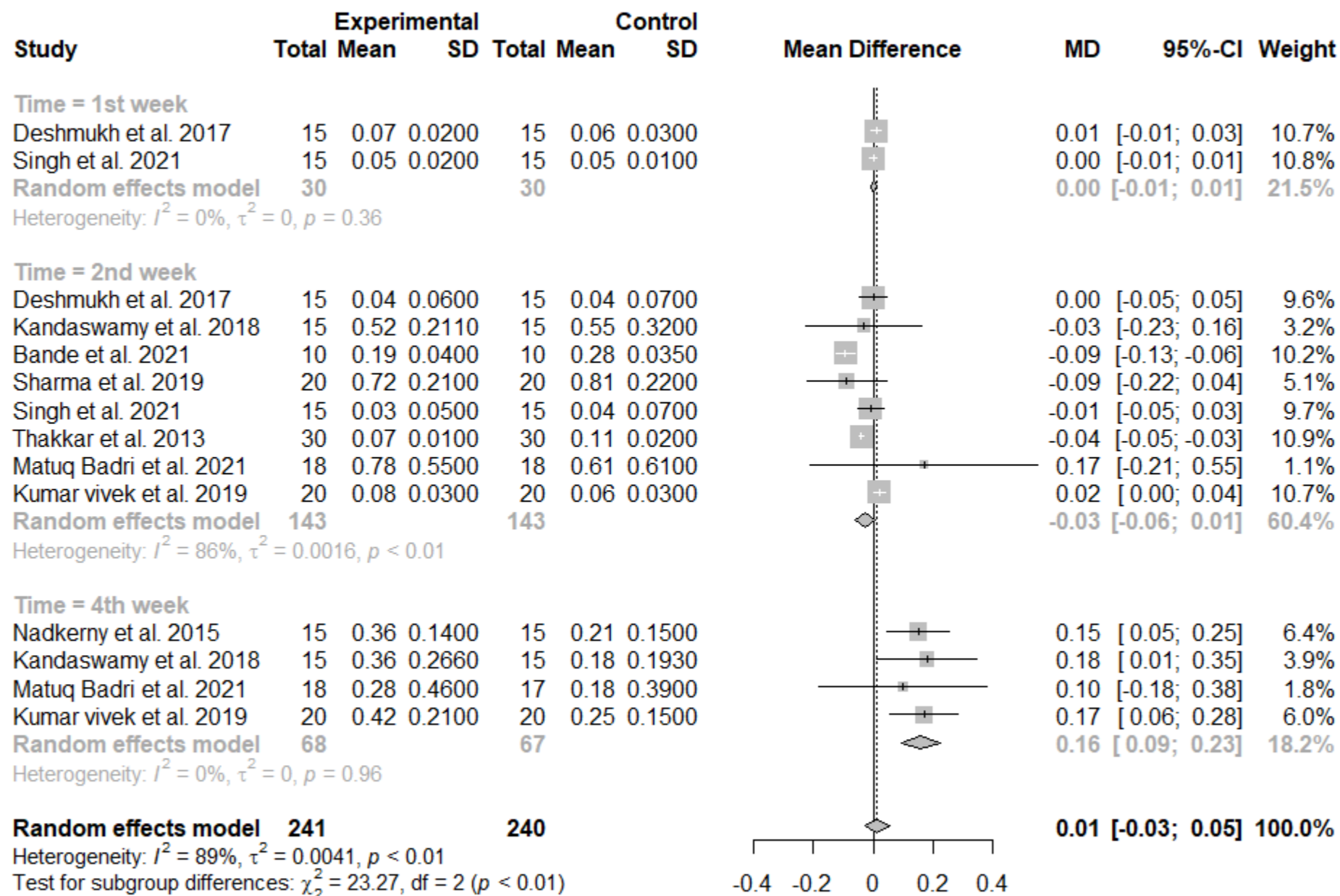

Supplement: Supplementary Materials — Supplementary Figure 1. Detecting outlier studies for the gingival index. Supplementary Figure 2.Meta-analysis after removing outlier studies for the gingival index. Supplementary Figure 3. Detecting outlier studies for the plaque index. Supplementary Figure 4.Meta-analysis after removing outlier studies for the plaque index. Supplementary Table 1. Databases and search strategy. Supplementary Table 2. GRADE certainty of the evidence. [file 4013004.f1.zip › Supplementary figure 4.pdf]
